# Alcohol Affects the Brain's Resting-State Network in Social Drinkers

**DOI:** 10.1371/journal.pone.0048641

**Published:** 2012-10-31

**Authors:** Chrysa Lithari, Manousos A. Klados, Costas Pappas, Maria Albani, Dorothea Kapoukranidou, Leda Kovatsi, Panagiotis D. Bamidis, Christos L. Papadelis

**Affiliations:** 1 Lab of Medical Informatics, Medical School, Thessaloniki, Greece; 2 Laboratory of Physiology, Department of Physiology and Pharmacology, Medical School, Thessaloniki, Greece; 3 Department of Forensic Medicine and Toxicology, Medical School, Thessaloniki, Greece; 4 Department of Neurology, Boston Children's Hospital, Harvard Medical School, Waltham, Massachusetts, United States of America; University College of London - Institute of Neurology, United Kingdom

## Abstract

Acute alcohol intake is known to enhance inhibition through facilitation of GABA_A_ receptors, which are present in 40% of the synapses all over the brain. Evidence suggests that enhanced GABAergic transmission leads to increased large-scale brain connectivity. Our hypothesis is that acute alcohol intake would increase the functional connectivity of the human brain resting-state network (RSN). To test our hypothesis, electroencephalographic (EEG) measurements were recorded from healthy social drinkers at rest, during eyes-open and eyes-closed sessions, after administering to them an alcoholic beverage or placebo respectively. Salivary alcohol and cortisol served to measure the inebriation and stress levels. By calculating Magnitude Square Coherence (MSC) on standardized Low Resolution Electromagnetic Tomography (sLORETA) solutions, we formed cortical networks over several frequency bands, which were then analyzed in the context of functional connectivity and graph theory. MSC was increased (p<0.05, corrected with False Discovery Rate, FDR corrected) in alpha, beta (eyes-open) and theta bands (eyes-closed) following acute alcohol intake. Graph parameters were accordingly altered in these bands quantifying the effect of alcohol on the structure of brain networks; global efficiency and density were higher and path length was lower during alcohol (vs. placebo, p<0.05). Salivary alcohol concentration was positively correlated with the density of the network in beta band. The degree of specific nodes was elevated following alcohol (vs. placebo). Our findings support the hypothesis that short-term inebriation considerably increases large-scale connectivity in the RSN. The increased baseline functional connectivity can -at least partially- be attributed to the alcohol-induced disruption of the delicate balance between inhibitory and excitatory neurotransmission in favor of inhibitory influences. Thus, it is suggested that short-term inebriation is associated, as expected, to increased GABA transmission and functional connectivity, while long-term alcohol consumption may be linked to exactly the opposite effect.

## Introduction

Social drinking, for most people, is an inseparable part of every-day life. Alcohol is used and abused for its ability to modify emotional states, and more precisely, to reduce anxiety [1], [2]. It is therefore essential to study the effects of inebriation in healthy, non-dependent individuals, given the frequency of abuse and binge drinking. A better understanding of the neural underpinnings of alcohol consumption could have a number of social implications, including driving situations, work-related hazards and others [3].

Acute alcohol administration affects behavior [4], [5] cognition [6], [7], and affective stimuli processing [8], [9]. It is responsible for the attention deficit observed even in low or moderate levels of inebriation [8], [10], [11]. The neurophysiological mechanisms by which alcohol acts on the brain modifying behavior have received much attention from scientists especially during the last few decades. However, they are still poorly understood.

An important finding is that short-term alcohol consumption disrupts the delicate balance between inhibitory and excitatory neurotransmission in favor of inhibitory influences [12], [13]. Inhibitory neurotransmitters transiently decrease the responsiveness of other neurons to further stimuli, whereas excitatory neurotransmitters produce the opposite effect. Evidence suggests that alcohol can acts by increasing inhibitory neurotransmission, by decreasing excitatory neurotransmission, or through a combination of both [13]. The general consensus is that acute alcohol intake facilitates the GABAergic transmission, and inhibits glutamatergic function [14]. The transmission and function of other neurotransmitters like dopamine, serotonin and opiates is also affected.

Alcohol's effect on neurotransmitters may, at least partially, explain some of the complex behavioral manifestations of intoxication in both animals and humans. GABA induction has been associated with the observed sedation effects [15], [16] of alcohol, the reinforcement of dopaminergic [17] and opiate pathways [18] are responsible for the excitement following alcohol administration, and the inhibition of the glutamate activity [19] is linked to difficulty in the formation of new memories and muscular coordination [20].

The GABA neurotransmission is of particular interest in alcohol intoxication, since (i) it has been associated with a person's susceptibility to develop alcohol abuse or dependence [21], (ii) it is the main inhibitory neurotransmitter in the human brain, and (iii) its receptors are the most common in the mammalian nervous system [22]. Alcohol inhibits the activity of signal-receiving neurons by interacting with the GABA_A_ receptor embedded in their cell membrane [23]. When GABA molecules bind to the receptor and activate it, the latter temporarily opens and allows the entrance of negatively charged molecules, such as chloride ions. Alcohol enhances the ions flow into cells thereby enhancing neuronal inhibition.

The disrupted balance between inhibitory and excitatory neurotransmission at the microscopic level caused by alcohol intoxication, may be reflected at the macroscopic level in the brain activity recorded even from outside the human scalp. Recent electroencephalographic (EEG) findings suggest that neurotransmission inhibition, induced by GABAergic agonist lorazepam, can cause a widespread increase in the inter-area functional connectivity and an increase in the strength of functional long-range and inter-hemispheric connections [24]. Thus, it was proposed that enhanced inhibition is an efficient mechanism for the synchronization of large neuronal populations in the human brain. The neural synchronization is regarded as a candidate neurophysiological mechanism to explain the dynamic interaction between spatially distributed regions ([25], see [26] for a review on this topic).

Since alcohol admission affects significantly the inhibitory function of GABA_A_ receptors and enhanced inhibition increases the inter-area functional connectivity, we hypothesize that short-term alcohol consumption will increase the functional connectivity of the human brain at rest. We expect the enhancement of the GABA inhibition activity to potentially affect the brain as a whole system rather than specific brain regions in particular, since GABA_A_ receptors are widely distributed in the human brain.

In the current piece of work, we examine how acute alcohol intake modulates the human brain functional coupling of the resting-state network (RSN). The study of RSN is of particular importance for studying the GABA neurotransmission mechanism, since GABA concentration in specific regions has been recently found to be positively correlated with their activation at rest [27]–[29]. To test our hypothesis, EEG data were recorded from non-addicted young individuals at rest after they have been administered with an alcoholic beverage or a placebo. Magnitude Square Coherence (MSC) was used to measure the functional connectivity between cortical sources estimated with standardized Low Resolution Electromagnetic Tomography (sLORETA) [30]. Since we expect large-scale brain connectivity to be affected, the global structural characteristics of the brain functional network should be studied. This kind of information is not available by routine functional connectivity analysis. Thus, as suggested in previous literature [31]–[33] as well, graph theory was employed for this analysis and the parameters that quantify the topological characteristics of the brain functional networks were estimated. Their modulation by alcohol was statistically tested to reveal information about the changes in the nature of brain networks due to short-term consumption of alcohol. Salivary alcohol and cortisol served to measure the inebriation and stress levels accordingly. The correlation of parameters of the brain functional network to the salivary alcohol and cortisol concentration were also tested.

## Materials and Methods

### Participants

Twenty-six healthy right-handed individuals (12 females, 14 males), with a mean age of 26.1±1.8 (mean ± SD), and free of any neurological or hepatic disorders, participated in the study. Female participants were in the first half of their menstrual cycle. All participants were free of any medication and completed a questionnaire on their drinking habits. The selected participants were characterized as light drinkers [34], meaning that they were accustomed to drinking 1–3 drinks per occasion, 1–2 times weekly. Their Body Mass Index (BMI) was 21.8±3.1 (mean ± SD). Participants were asked to refrain from alcohol consumption for at least 48 hours prior to recordings, as well as from smoking, caffeine, and food for at least 3 hours prior to recordings. The experimental procedure was approved by the ethical committee of the Medical School of the Aristotle University of Thessaloniki. All volunteers provided written informed consent prior to experimentation.

### Experimental design

Each participant was tested in two experimental sessions and received a real alcohol dose (alcohol session) and a placebo beverage (placebo session). The order of the sessions was counterbalanced, while the doses were administered double-blindly. Thus, half of the participants had an alcoholic beverage in the first session and a non-alcoholic placebo beverage in the second, while the order was reversed for the other half. The two sessions were performed at least six weeks apart to ascertain that the participants had no clear memory of the first session inebriation level. All experiments started at the same time of the day (16.00) in order to simulate as close as possible real life situations (people consume alcohol usually late in the afternoon and/or evening) and to avoid differences in the circadian rhythm. EEG and electrooculography (EOG) were collected for five minutes during an eyes-open as well as an eyes-closed session. During eyes-open session participants were asked to fixate on a white cross on a black screen placed 80 cm in front of their eyes, relax, and refrain from blinking and moving their eyes to the extent possible. They were also advised to keep their head still and refrain from falling asleep.

### Beverage manipulation

During the alcohol session, participants drank a mixture of two parts of orange beverage and one part of vodka (vol 37.5%). The mixture was divided in four doses, each consumed within 5 min. As a result, alcohol administration lasted twenty minutes and was followed by an absorption period of 25 min. The alcohol dose was adjusted (for males 0.48 g/kg and for females 0.38 g/kg) in order to achieve a Blood Alcohol Concentration of 0.7 g/L [35]. During the placebo session, the same volume of drink, consisting only of orange beverage, with a few drops of vodka on the surface and on the glass edge to achieve the odor of alcohol was offered to the participants. The first dose contained slightly more vodka drops than the following ones, in order for the participants to sense a strong initial taste of alcohol similar to the alcoholic beverage [36], [37].

### Saliva alcohol concentration

In order to assess inebriation following acute alcohol intake, saliva samples were collected [38], [39] 20 min after the end of the alcoholic or the placebo beverage administration. The above time was selected since peak alcohol blood concentration is reached twenty minutes following consumption [40]. The Saliva Alcohol Concentration (SAC) was determined by head-space gas chromatography coupled with a flame ionization detector. All analyses were performed with an in-house method previously developed and fully validated in [41]. The SAC achieved was 0.665 g/L±0.17 and no significant difference was observed between males and females (p = .875, F (1, 25) = .026). Following consumption of the placebo beverage, no alcohol was detected in saliva.

### Saliva cortisol concentration

Two extra saliva samples were collected from each participant during each session in order to measure salivary cortisol. The first one was collected upon arrival, prior to the procedure (pre-intake), while the second one 20 min after beverage consumption (post-intake), just before the recording, when alcohol is expected to reach its peak concentration in blood [40] and cortisol level is expected to be modulated [42].

The Salivette device (Sarstedt, Numbrucht) was used to collect and measure the salivary cortisol. The sample was moved into the laboratory's cooler and it was centrifuged in a cylindrical plastic tube to isolate the saliva, approximately 1.5–2 mL. Saliva samples were then stored at −20°C. The participant must abstain from food, and coffee at least two hours prior to sampling, as these factors change the environment of the oral cavity and the activation of the Hypothalamic Pituitary Adrenal (HPA) axis.

The measure of the cortisol in the saliva samples was performed with the Enzyme-Linked immunosorbent test (ELISA), also known as immunoassay, which is mainly used in immunology to detect the presence of an antibody or an antigen in a biological sample. The lower limit of sensitivity was determined by interpolating the mean minus 2 standard deviations for 18 zero standards. The minimal concentration of cortisol that can be distinguished from zero, is 2.5 pg/mL.

The difference in the stress levels caused by alcohol or placebo intake was assessed indirectly, that is, by means of the difference between the post-intake and the pre-intake cortisol values. The difference in the stress levels of the participants was the same for alcohol and placebo administration (p = .5, F (1, 25) = .046), meaning that the effect of alcohol and placebo drink on the stress levels was the same, as in previous literature [34], [43].

### Data recordings and preprocessing

EEG signals were recorded (Nihon Kohden) from 57 scalp sites with a sampling rate of 500 Hz. The scalp electrodes were placed on a cap (EASYCAP) according to the 10/10 system. The electrodes were commonly referenced to the average of the two linked mastoid electrodes. EOG signals were also recorded simultaneously by means of four electrodes placed around the eyes (one above and one below the right eye, and another two placed at the outer canthi of each eye). The vertical EOG (VEOG) was calculated as the difference between the two signals recorded above and below the right eye, while the horizontal EOG (HEOG) as the difference between the signals recorded from the left and the right electrodes respectively. EEG signals were band-pass filtered between 0.5 and 90 Hz, while EOG signals between 0.5 and 8 Hz. Ocular artifacts were removed from the EEG signals by means of the REG-ICA methodology applying the technique proposed in [44]. From each dataset, ten (10) sec of ongoing brain activity were randomly selected by two individual experts to be included in the analysis. The artifact free segments were selected by two independent EEG experts in our lab. Each of them had to select 15 segments of artifact-free EEG segments from a continuous recording of 10 min. An overlap of at least ten segments between the two experts was required in order for the data to be further used in the analysis. In case there was a discrepancy between the selected EEG segments from the two experts, the common segments were used and completed by the artifact-free EEG segments selected by the first expert.

### Estimation of cortical sources

Cortical source time series were estimated with sLORETA [30] using Brainstorm 3.0 ([45], http://neuroimage.usc.edu/brainstorm). The sLORETA solutions are computed within an average head model reconstructed of 152 normal MRI scans (Montreal Neurological Institute, http://www.loni.ucla.edu/ICBM/) co-registered to the Talairach probability brain atlas [46]. The four basic layers (scalp, outer-skull, inner-skull, and cortex) which describe the different conductivity levels of the head structures [47], [48] were extracted using the Boundary Element Method (BEM), implemented in the Brainstorm toolbox. The different compartments of the volume conductor model were approximated by fitting a closed triangular mesh with 259 nodes onto the cortex, in order to get a smooth approximation of the average cortical surface (131 at left and 128 at right). On each vertex there is only one dipole and its orientation is the normal to the cortex surface at that point. Thus, the cortical network was down-sampled to the ‘a-priori’ defined 259 electrical dipoles-nodes. The electrode coordinates were based on the average location of the 10-20 EEG system. Selected artifact-free EEG segments were used for calculating the sLORETA intracranial spectral density with a resolution of 1 Hz, from 1 to 45 Hz.

### Functional connectivity analysis

Functional connectivity was estimated across cortical sources, rather than across EEG sensors, because source topographies largely overlap at the EEG sensor level. We used MSC as a pair-wise connectivity measure.

MSC is a function of the Power Spectrum Density (PSD) of two signals x and y (P_xx_ and P_yy_), and the cross PSD (P_xy_) of signals x and y in a certain frequency f. 
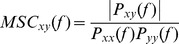



P was estimated using the Welch method [49]. The signals were divided into segments containing 400 samples, with 50% sample overlap. P was then computed using the formula:
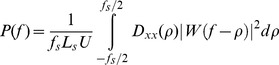
where 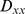 is the Discrete Fourier Transform of the signal's correlation sequence 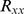, 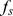 is the sampling frequency (here 500 Hz), 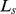 is the segment length and 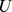 is a normalization constant ensuring that P is asymptotically unbiased. 
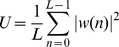



Finally, the 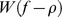 is a Hamming window centered on frequency 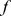
_._ The MSC of each pair of cortical dipoles, x and y, was calculated for each frequency band (delta (δ) 0.5–4 Hz, theta (θ) 4–8 Hz, alpha (α) 8–12 Hz, beta (β) 12–30 Hz, gamma (γ) 30–45 Hz).

The networks consisting of nodes and connections were, therefore, mathematically represented as graphs. This information was stored in the form of a 259×259 Adjacency Matrix (AM), which in this case is symmetric, since MSC_xy_  =  MSC_yx_. For each subject, two weighted AM matrices were calculated, one for the alcohol and another for the placebo session. To reduce the complexity of the AMs, the original MSC values ranging from 0 to 1 were subjected to subsequent thresholds converting the weighted graphs into binary ones.

### Calculated graph parameters

For each threshold and frequency band, for each of the two binary networks (alcohol and placebo sessions), and for each participant, the following parameters were calculated: density, characteristic path length, global efficiency, clustering coefficient, small-worldness and degree of each node.

Density (K) is the simplest parameter and is defined as the number of existing edges (E) divided by the number of the possible edges among nodes (N).
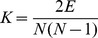



Characteristic path length (L) is an integration measure and is defined as the average of the shortest paths L_i_ between each pair of connected nodes. 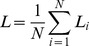



Global efficiency (E) is also an integration measure and is defined as the arithmetical mean of the inverse of the shortest paths between each pair of nodes.
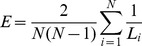



Clustering coefficient (C) is a segregation measure quantifying the trend of the network to form clusters. It is defined as the mean of the C of each node i. The C_i_ of each node is the proportion of links between the vertices within its neighborhood divided by the number of links that could possibly exist between them.
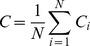



Characteristic path length, global efficiency and clustering coefficient were normalized (divided) by the corresponding parameters of random surrogate graphs [50].

The small-worldness (S) of a network is defined as the ratio of the clustering coefficient (C) divided by the characteristic path length (L), both normalized by a set of random graphs with the same number of nodes and connections [51]. A network is characterized as a small-world one, when S>1), meaning that the network has a significantly higher clustering coefficient (C) than a corresponding random one, while it has approximately the same characteristic path length (L) as a corresponding random one.
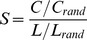



Finally, in an undirected network, the node degree is defined as the number of links connected to this node. This is not a large-scale parameter, since it is calculated for each node separately (herein 259) and it is not one single value globally characterizing the network.

### Statistical analysis

For each connection during both eyes-open and eyes-closed sessions and in all frequency bands, repeated measures Analysis of Variance (ANOVA) was performed on the MSC values to reveal differences (alcohol vs. placebo intake) in the connectivity. The results were corrected for multiple comparisons (False Discovery Rate (FDR) [52]).

After constructing the binary graphs for multiple thresholds, repeated measures ANOVA (alcohol vs. placebo) was performed for each threshold on the globally estimated network parameters (density, characteristic path length, global efficiency and clustering coefficient, p<.05) and on the degree of each node (p<.05, FDR corrected similarly to [53]).

Correlations of the inebriation level, measured through SAC, with the concurrent network parameters during alcohol sessions were calculated for each threshold and only for the frequency bands where significant alcohol effects were observed. The post-intake cortisol values, reflecting the stress levels at the time of EEG recordings, were also correlated with the concurrent network parameters in all frequency bands for both the alcohol and placebo sessions. Pearson's correlation was used and the significance level was set to .05.

Finally, it was tested for all thresholds whether the functional networks obtained during alcohol and placebo sessions contrast significantly to ‘random’ graphs or not. A set of 1000 random undirected graphs with the same number of nodes and links as our experimental graphs were generated (similarly to [54]). Z-tests were performed at 0.05 (FDR corrected) to detect significant differences between the average global and local efficiency values between the experimental graphs and the random ones.

All statistical analyses were performed in Matlab R2010a (MathWorksInc, USA).

## Results

### Effect of alcohol intake on connectivity

**Figure 1:**
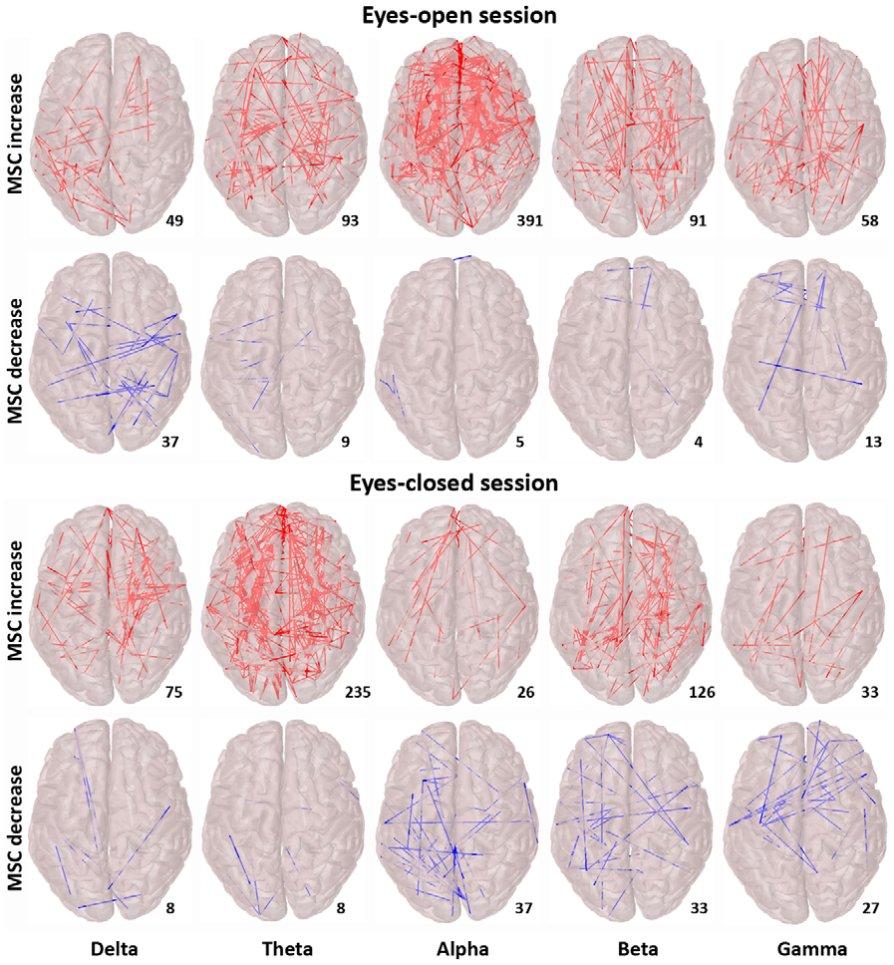
Alcohol-induced significant connectivity differences. Differences during eyes-open and during eyes-closed sessions are presented in the upper and lower panel respectively. Significantly (p<.05, FDR corrected) increased connectivity is shown in the upper rows (red connections), decreased in the lower rows (blue connections). The number of significantly affected connections is also presented for each frequency band.

Figure 1 presents the alcohol-induced MSC increase and decrease after alcohol intake (vs. placebo) for each frequency band during sessions with eyes-open or eyes-closed. Large-scale functional connectivity was increased for the alpha and theta bands for eyes-open and eyes-closed sessions respectively. A significant decrease of MSC was also observed, but only on a limited number of connections. The highest number of significantly affected connections was observed in the alpha band (391 increased vs. 5 decreased) for the eyes-open session and in the theta band (235 increased vs. 8 decreased) for the eyes-closed session.

### Effect of alcohol intake on graph parameters

**Figure 2:**
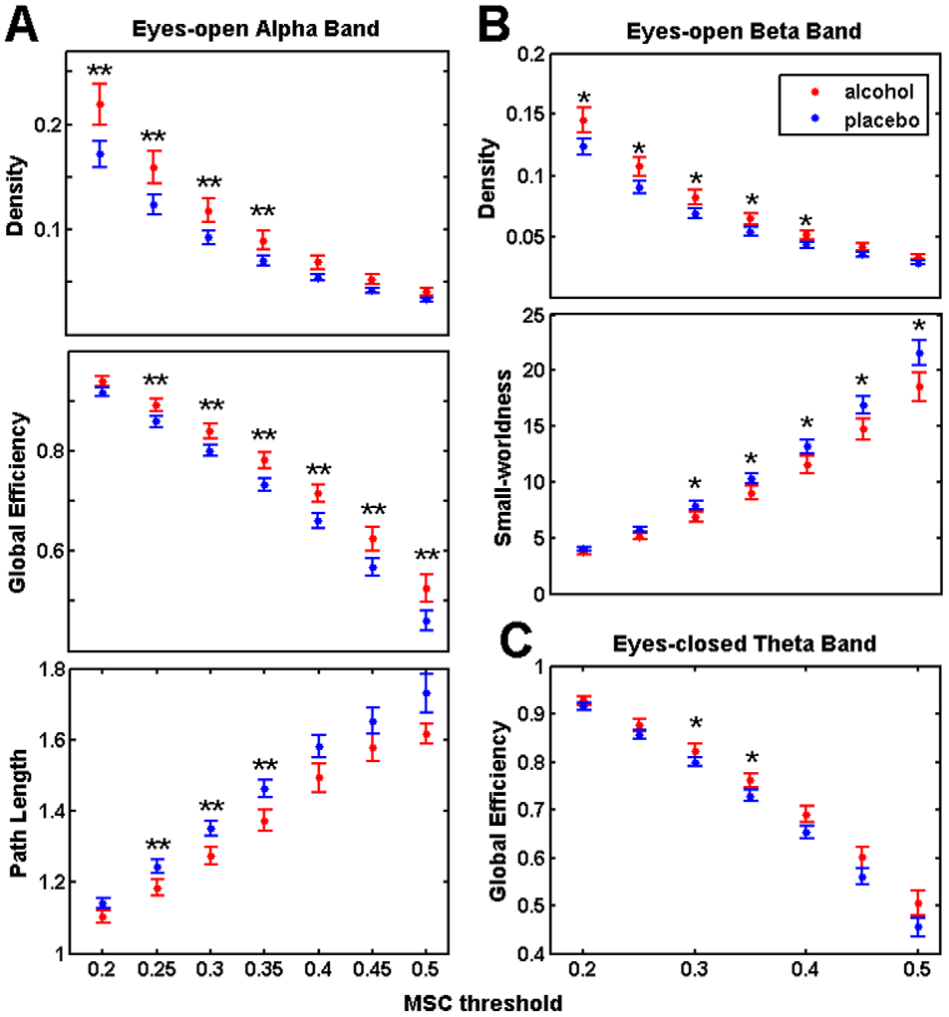
Significant alcohol effect on graph parameters across different thresholds. Double stars (**) indicate significant p-values (p<0.03), single stars (*) indicate marginally significant effects (p<0.06). To maintain the high resolution and quality of the figures, we did not include all the results for the many different thresholds, but only for the first seven. Characteristic path length, clustering coefficient, and consequently small-worldness are normalized by random surrogate graphs [49].

The graph density, path length, global efficiency and clustering coefficient were compared across participants between alcohol and placebo sessions. Figure 2 illustrates all these graph parameters and their modulations by alcohol across different thresholds.

In alpha band, density and global efficiency were higher and characteristic path length was lower during the alcohol session as compared to placebo (see Figure 2A). In beta band, graph density was higher, while small-worldness was lower during the alcohol (see Figure 2B); however, these effects were marginally significant (p = 0.06). Marginally significant statistics are also reported since they were observed across subsequent thresholds. Global efficiency was also marginally increased during alcohol in the theta band (p = 0.058) for the eyes-closed session (see Figure 2C). We did not observe any significant difference in other frequencies or in other graph parameters. At threshold 0.35, all observed alcohol effects were significant (or marginally significant). For clarity purposes, we have, therefore, selected to show the rest of the results for this representative threshold.

**Figure 3:**
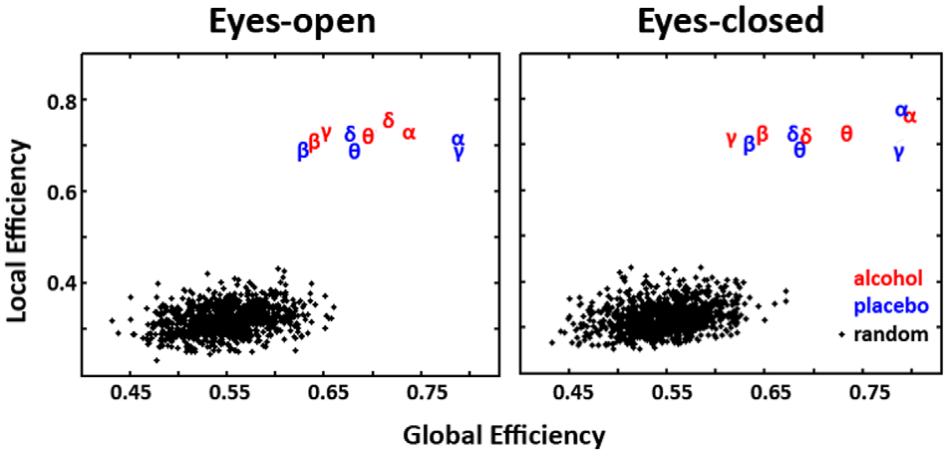
Scatter plot of global and local efficiency (threshold set to 0.35). Averaged values are grouped for alcohol (red symbols) and placebo (blue symbols) for both eyes-open (left panel) and eyes-closed (right panel) sessions. Greek letters designate the frequency band studied accordingly. The corresponding values of 100 random graphs are represented with black dots.

Figure 3 illustrates the contrast between random graphs and those obtained and treated in this study. Separate z-tests were performed to test the statistical significance of the differences in the mean values of global and local efficiency and the z-scores are summarized in Tables 1 and 2 respectively. It is evident that for both alcohol and placebo sessions in all frequency bands, global efficiency and local efficiency of the obtained graphs are significantly higher than the random ones. This fact indicates clearly that the graphs obtained and treated in this analysis are not random ones.

**Table 1:** Contrast of eyes-open graphs with random surrogates.

Eyes-open session										
z-scores	Alcohol					Placebo				
	δ	θ	α	β	γ	δ	θ	α	β	γ
Global Efficiency	145.82	141.23	152.7	128.56	130.32	138.13	138.83	167.29	125.15	167.28
										
Local Efficiency	223.17	217.42	231.78	201.56	203.76	213.53	214.41	250.07	197.28	250.06

z-values of the 20 contrasts performed with a z-test with significance level at 0.05 (FDR corrected). All contrasts were significant (p<0.001).

**Table 2:** Contrast of eyes-closed graphs with random surrogates.

Eyes-closed session										
z-scores	Alcohol					Placebo				
	δ	θ	α	β	γ	δ	θ	α	β	γ
Global Efficiency	145.96	141.36	152.84	128.7	130.46	138.26	138.96	167.44	125.29	167.43
										
Local Efficiency	212.94	207.45	221.16	192.32	194.42	203.75	204.58	238.61	188.24	238.6

z-values of the 20 contrasts performed with a z-test with significance level at 0.05 (FDR corrected). All contrasts were significant (p<0.001).

**Figure 4:**
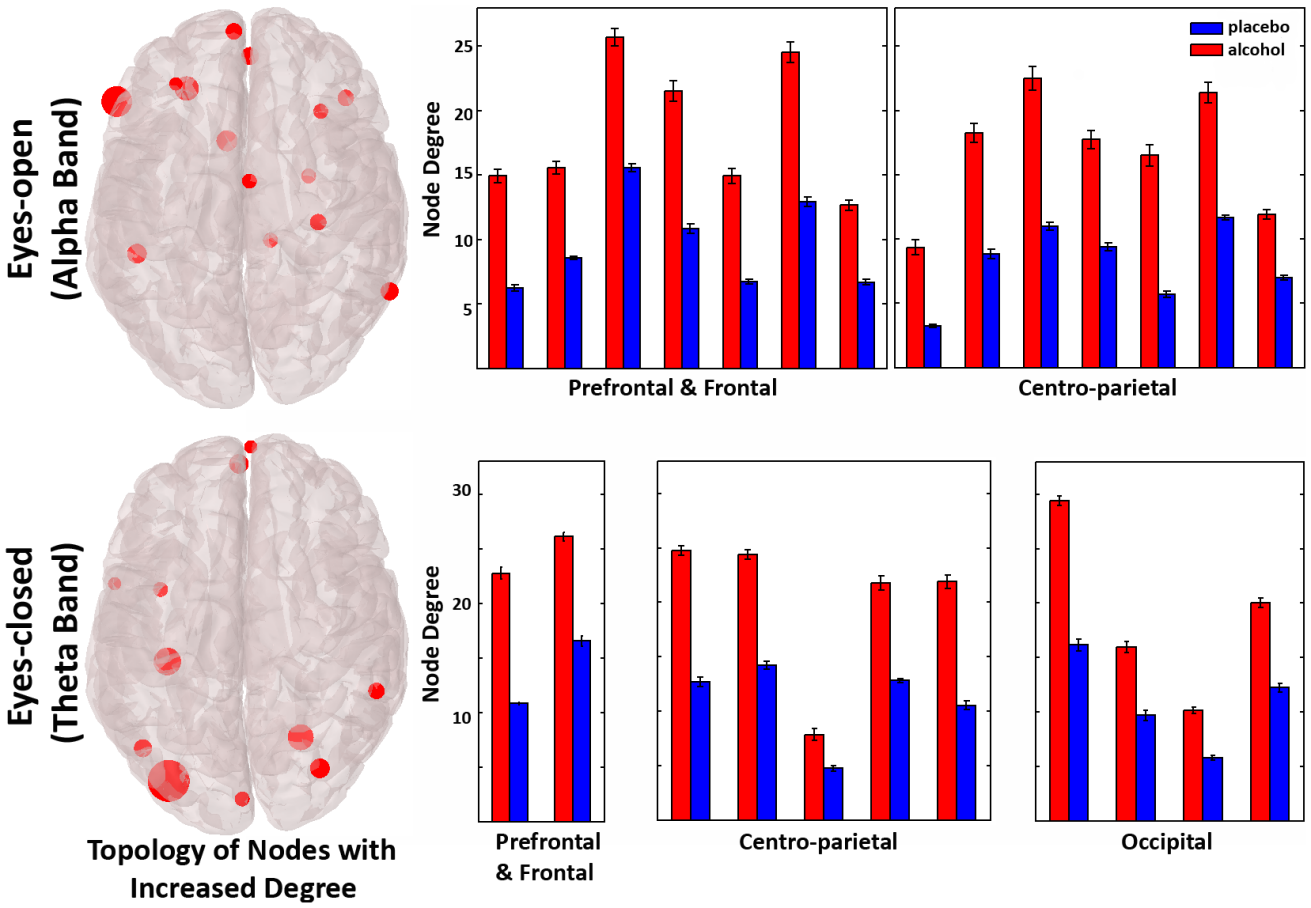
Topologies of the nodes with increased degree following inebriation for a representative threshold of 0.35. Left: The highlighted nodes have a degree significantly altered by alcohol intake (vs. placebo) in alpha band during eyes-open and in theta band during eyes-closed. The size of red nodes is inversely proportional to the significant (< .05) p-values: the larger the node the more significant the effect is. Right: Mean node degree values of the nodes significantly affected (p<.05) by alcohol for alpha band (upper row) and theta band (lower row) networks averaged across participants (± SD).

Figure 4A presents the effect of alcohol on node degrees during eyes-open in alpha band. For specific nodes located mainly in anterior and medial sites, we observed significant increase of their degree (p<0.05, FDR corrected). The effect of alcohol on node degrees during the eyes-closed session in the theta band is presented in Figure 4B. A significant increase of node degree (p<0.05, FDR corrected) was observed in specific nodes located mainly in posterior and medial sites (see Figure 4B). No significant effects of alcohol were observed for node degrees in any other frequency band.

### Correlation of SAC and cortisol levels with the RSN parameters

**Figure 5:**
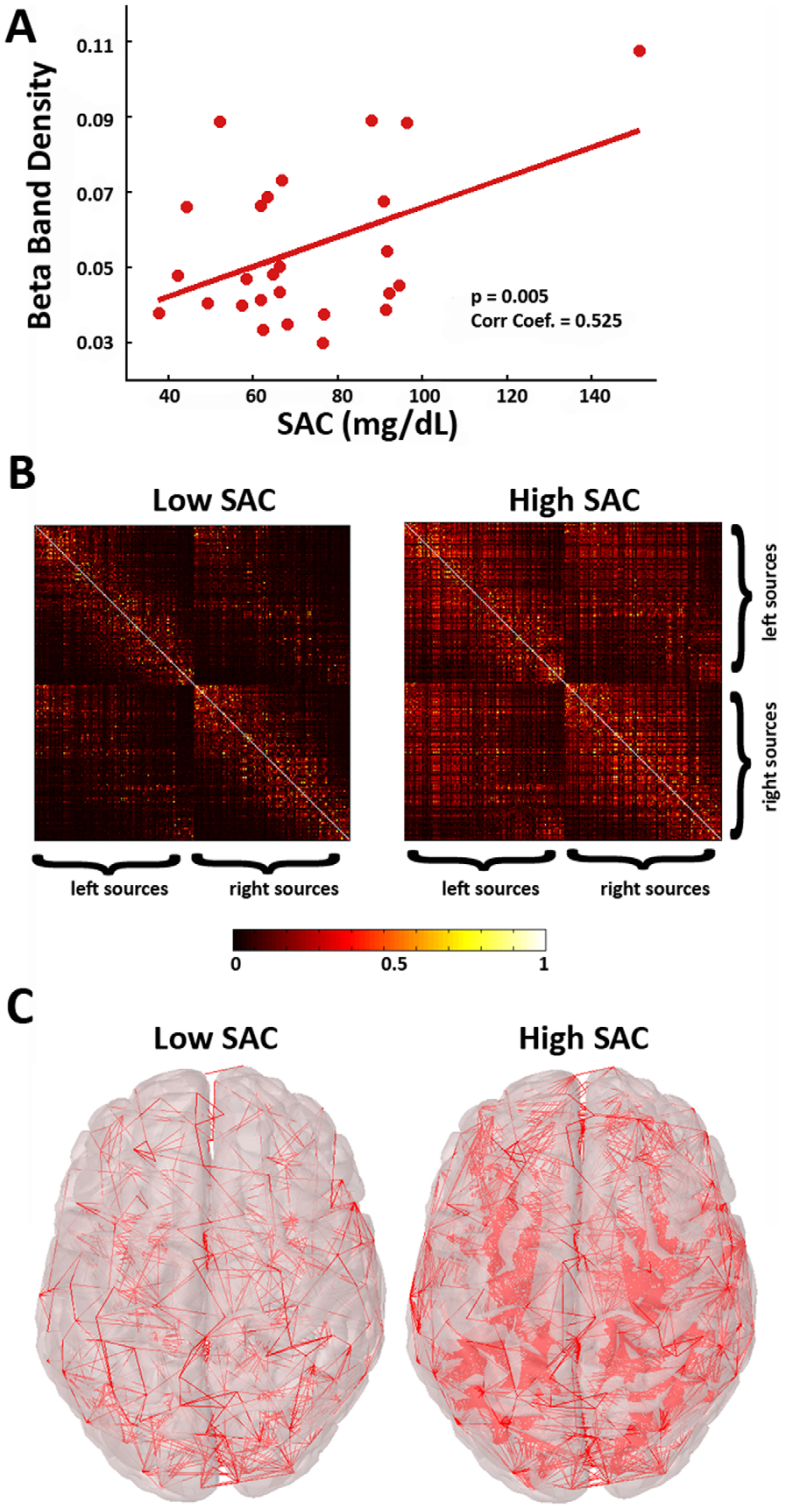
Correlation of SAC with the density of the networks for beta frequency band. (A) Scatter plot of SAC values versus beta band density during the alcohol session; (B) To illustrate this correlation, the averaged functional networks of the 7 participants with the lowest and 7 participants with the highest SAC values are presented as weighted AMs. The weights of these AMs are the MSC values; (C) connections of the networks in (B), after being converted to binary graphs using a representative threshold of 0.35.

Since alcohol affected only the network parameters on theta (eyes-closed), alpha and beta (eyes-open) frequency bands, possible correlations of network parameters with SAC and cortisol levels were examined selectively for these bands across different thresholds. The correlation between the network parameters and SAC was estimated only during the alcohol sessions, since no alcohol was detected in the saliva samples in placebo. Significant (p<0.01) positive correlations between SAC and density for thresholds from 0.2 till 0.5 were observed in beta band. Figure 5 illustrates the correlation for the particular threshold of 0.35. Figure 5B and 5C show the functional networks of participants with high and low SAC. Cortisol concentration in the saliva was not correlated with any graph parameters in any frequency band or eye session either.

## Discussion

In the present study, the neurophysiology of short-term inebriation was studied for the first time under the context of functional connectivity and graph theory. Based on the already reported (i) facilitated large-scale functional connectivity due to enhanced GABAergic transmission [24], and (ii) the alcohol-induced enhancement of GABA inhibition [13], [14], we hypothesized that the acute intake of a moderate dose of alcohol would increase the brain functional connectivity at rest in social drinkers. Our hypothesis that the enhancement of the inhibition activity will affect the whole brain as a functional network, rather than specific regions in particular has been confirmed by our findings. The functional connectivity was indeed elevated following inebriation in theta, alpha and beta bands, while the RSN parameters were accordingly affected. Density and global efficiency were higher, while path length was lower following alcohol as compared to placebo. Higher inebriation levels, measured through salivary alcohol concentration, were linked to higher RSN density and specific nodes exhibited higher degree following inebriation.

### Alcohol increased the functional connectivity of the RSN

Our hypothesis was confirmed by the elevated functional connectivity in alpha, beta and theta bands after alcohol as compared to placebo. Increased undirected functional connectivity (herein estimated with MSC) is indicative of increased functional coupling between distributed brain regions exhibiting synchronized neuronal activity [55]–[57]. The synchronization of large neuronal populations in alpha and beta bands during rest has been attributed to neural inhibition, through GABA-signaling [24]. Alcohol is known to induce neural inhibition by enhancing the function of GABA_A_ receptors [58]. The observed increased functional connectivity after short-term alcohol consumption may be due to the disrupted balance between inhibitory and excitatory neurotransmission towards enhanced GABA-induced inhibition. It was assumed that this process may be governed by voltage-dependent mechanisms in the neuronal somas and the strategic location of inhibitory synapses on the somas [59], [60]. Both specific synaptic and diffuse extra-synaptic GABA_A_ receptors, which are highly distributed in the human brain, may mediate the large-scale synchrony involving coordination between localized networks.

The increased functional connectivity after short-term alcohol intake is further supported if one considers the reported reduced functional connectivity observed in chronic alcoholic patients [61]. The balance between inhibitory and excitatory neurotransmission is disrupted in favor of inhibitory influences after short-term alcohol consumption [12], [13]. However, evidence suggests that after long-term alcohol consumption the brain attempts to restore the equilibrium. Thus, although short-term alcohol consumption may increase GABA_A_ receptor function, prolonged drinking has the opposite effect [23], [13]. A decrease in the GABA_A_ receptor function will thus be reflected as reduced functional connectivity in chronic alcoholic patients, as it has already been shown by Rogers and colleagues [61]. On the other hand, an increase in the GABA_A_ receptor function will be reflected as increased functional connectivity after short-term alcohol consumption.

We should state, however, that since acute alcohol intake affects multiple neurotransmitter systems, like the dopaminergic and the opioids' [15]–[20], we cannot exclusively attribute the increased connectivity to the specific neurophysiological mechanism of enhanced GABA transmission. A potent link between these systems and functional connectivity in the human brain remains to be further investigated.

### Alcohol-induced connectivity in the alpha, beta, and theta bands

The majority of the significant alterations in MSC values were reported as MSC increases (vs. MSC decreases) observed almost in all frequency bands; however, they were more prominent in the theta, alpha and beta (see Figure 1) bands. The importance of alpha band in the synchronization of the human RSN has been reported by both functional mapping [62]–[64] and electrophysiological studies [65]–[67]. Increased alpha synchronization at rest is observed in those brain regions that are deactivated during attention demanding tasks [63], [64]. The alcohol-induced attention deficit [8], [10], [11] can be traced back to pronounced alpha band functional connectivity, which indicates a relaxing inhibited state of low attentional demands [64] [66]. When attention is required, the human brain under short-term inebriation has to perform a transition from a supposedly ‘deeper’ resting state towards awareness. Moreover, functional connectivity in beta band, although less studied, has been reported at rest [65], [68]. Decreased functional connectivity at rest is coupled with low beta band power [69]. Lower resting-state functional connectivity in beta and alpha bands has been linked to pathology [70], [71]. Interestingly, in [72] it is deduced that alpha and beta oscillations are coalescenced at rest.

The role of theta band at rest is also prominent; sharper topography of the DMN was obtained in alpha and theta bands in MEG recordings [73]. There is also a study reporting a positive activation in theta band in the entire cortex in eyes-closed resting state, while this activation is diminished when the eyes are open [74]. This fact is in line with our results of increased connectivity in theta band mainly during eyes-closed sessions. Concluding, theta, alpha and beta oscillatory activities are those to contribute more in the RSN [73], a fact that explains the significance of our functional connectivity findings selectively in these bands.

### Alcohol affected the graph parameters of the RSN

The core graph parameters reflected also the elevated functional connectivity by detecting consistent alterations in the structure of brain complex networks. During eyes-open sessions, density and global efficiency were higher, while path length was lower following alcohol as compared to placebo in alpha band (see Figure 2A). The simultaneous increase of density and global efficiency, along with the decrease of path length, implies extra functional links among distant brain regions. This would facilitate their communication and pronounce a costly exchange of information, confirming our hypothesis for elevated large-scale connectivity.

Moreover, higher density accompanied with lower small-worldness was observed in beta band during eyes-open (see Figure 2B). This finding, although marginal, supports the significant positive correlation of density with inebriation levels measured in saliva. It is suggested that alcohol induces a prominent connectivity also in beta band. However, instead of a corresponding increase of efficiency (like in alpha band) the small-world structure seems to be obstructed indicating a meaningless increase of the total wiring cost in the brain network (see next section for more details).

During eyes-closed sessions, the global efficiency of the networks in theta band was marginally higher as compared to placebo (see figure 2C) suggesting again a facilitated distant communication. This finding is in line with a previous study reporting positive activation of the whole cortex in theta band during eyes-closed and not during eyes-open [74].

Core graph parameters were selected for this study since the main types of graph structure have been defined on the basis of their values [75]. Significant differences in graph parameters were observed in the bands with more pronounced connectivity increases (see Figure 1), that is alpha and beta for eyes open and theta for eyes-closed sessions. The main alcohol effects were significant at relatively low thresholds, where the obtained graphs had enough connections allowing the meaningful estimation of graph parameters and statistical comparisons between them [76].

### Inebriation levels were positively correlated to the RSN density

The most interesting finding of the present study is probably the correlation of alcohol concentration measured in saliva with the human RSN parameters. SAC is an accurate estimator of Blood Alcohol Concentration (BAC) [38], [39] and was used to objectively measure the inebriation level of the participants. SAC was found to be positively correlated with the density of the brain networks in the beta band (see Figure 5). A higher SAC was related to a higher number of functional connections in the network that corresponds to an increased total wiring cost [77]. So, higher inebriation levels indicate higher total wiring cost, which in the brain depends on the conduction delay along neural axons, the attenuation of signals in dendrites and the number of synapses [78]. The positive correlation of SAC with beta band networks' density is supportive to the marginally significant effects of higher density and small-worldness in beta band following alcohol (vs. placebo).

Neither the correlation with SAC, nor the main effect of alcohol was significant on global efficiency or characteristic path length. This fact indicates an alcohol-induced meaningless increase of the total wiring cost and the absence of a corresponding enhancement of the communication in the network. Our study provides evidence for an obstructed functional connectivity pattern due to alcohol in beta band, a band that has been also linked to emotion and cognition [79]. Significant increase in beta band connectivity at rest has been reported also in pathological situations [80].

### The graphs under study are not random

From Figure 3 and Tables 1 and 2, it becomes evident that the graphs obtained by our EEG experiment are not structured like random ones; this is true for all frequency bands, following both placebo and alcohol and during both eyes-open and eyes-closed sessions. On the contrary, they exhibit higher global and local efficiency, which are both characteristics of small-world networks. High global efficiency means efficient communication in the brain network, enhancing interactions between distant cortical sites. Local efficiency, on the other hand, is similar to the clustering coefficient, describing the efficient communication in functionally segregated brain regions. In fact, local efficiency appears slightly privileged with respect to global communication in the experimental graphs. Our findings indicate that resting state networks, either following alcohol or placebo intake, maintain their well-recognized small-world structure [81], [82].

The random graphs used as surrogates herein, were generated keeping the same number of nodes and connections. There are also other approaches that preserve also the same degree distribution [83]. However, apart from the much higher computational requirements, little to no difference between this approach and the usual random surrogates has been reported [49] when used for the normalization of the parameters obtained from experimental graphs.

### Higher degree was observed for specific nodes

Effects of alcohol on the Central Nervous System (CNS) are primarily mediated via GABA_A_ receptors, which are expressed across the brain. Different parts of the brain have, however, a higher affinity for different subunits [84]. A recent fMRI study examined alcohol-induced variances in regional GABA_A_ binding potentials in humans [85]. Alcohol affected mostly the connectivity of the visual cortex, the cerebellum, the anterior cingulated cortex (ACC) and the brainstem in relation to somatosensory and sensorimotor areas, with the most extensive effect observed in the connectivity between the posterior cingulated cortex (PCC) and the auditory and somatosensory areas. Moreover, in alcoholics, an increased synchrony in the dorso-lateral prefrontal cortex (DLPFC) was observed at rest in an attempt to remap the brain to compensate for alcohol-induced impairments [86]. Thus, despite the effect of alcohol on large-scale connectivity mainly attributed to enhanced GABA transmission, there is also evidence for the modulation of certain functional networks in the human brain.

Following our findings on increased large-scale connectivity, the degree of specific nodes was higher following alcohol (vs. placebo). Our hypothesis for increased connectivity was reflected on the higher strength of connections, which in turn resulted to a larger number of connections on specific nodes. During eyes-open this was the case for nodes located on the frontal and medial sites in alpha band, while during eyes-closed this effect was observed for nodes located mainly on medial and posterior sites (see Figure 4). Higher node degree indicates a more central role of the node in the communication among cortical sites.

The spatial pattern of the DMN consists of the regions which are deactivated during attention demanding tasks independently of the task, implying significant functionality during resting state (see Figure 1 at [87]). The DMN involves regions in the ventral medial prefrontal cortex, the dorsal medial prefrontal cortex, and the posterior medial and posterior lateral cortices [88]–[90]. The nodes with higher degree seem to overlap, at least to an extent, with these regions adding some topographical evidence to the increased functional connectivity following alcohol intake. We cannot though provide safe localization information given the low spatial resolution of EEG and the lack of anatomical information from the individual subjects' MRIs.

### Methodological Limitations

From a methodological point of view, our study presents some limitations. Our hypothesis, based on the effects of alcohol on GABA neurotransmission is supported by our findings. However, since EEG does not allow measuring the expected alcohol-induced alteration on the concentration of GABA in the brain, we cannot safely deduce a causality relationship between the observed pronounced functional connectivity and the neurotransmission effects. Moreover, alcohol studies face the problem of individual susceptibility and tolerance developed to the effects of alcohol. This effect can be critical when studying the intoxication process [5]. To overcome this problem, we used a sufficiently large sample (26 participants) and each participant served as his/her own control during the placebo sessions. Alcohol effects appear to be dose-dependent. Having administered a dose of 0.48 g/kg (0.38 g/kg) alcohol to male (female) participants, our findings are limited to moderate-dose alcohol effects.

Since individual MRI structural scans were not available, we used a BEM model in a standard brain for all EEG reconstructions. Combined with the inherently low spatial resolution of EEG, we cannot make detailed inferences on the anatomical origin of the recorded oscillations. The ideal experimental design for this study would also need the recording of the individual subject's MRIs and/or additionally magnetoencephalography (MEG) recordings at rest. MEG, as a technique, fundamentally avoids the limitations of EEG in functional connectivity analysis (i.e. possible leakage due to common reference for all channels) thereby providing also very good localization accuracy for the active sources [91], [92]. Coregistration of the inverse solutions, derived either from EEG or MEG data, with the individual subjects anatomies would provide crucial information about the exact anatomical location of the active sources, rather than the rough topological estimation provided herein. It would be also interesting, apart from the resting-state, to examine how short-term inebriation may affect the human brain functional connectivity during cognitive tasks or emotional perception.

Regarding the connectivity methodology, different methods have been proposed to select the threshold to be applied on weighted AMs to convert them into binary ones. We followed the approach of arbitrary threshold to test if the significant results were preserved across multiple connection densities. There is still a lot of debate around this issue and, as a consequence, there is no optimal method for the selection of the threshold to construct graphs, although different approaches have been suggested (see [50] for a review).

## Conclusions

The current study provides evidence that acute moderate alcohol doses in healthy young social drinkers increases the functional connectivity of the RSN. Such increase is evident in terms of MSC values and it is also reflected on graph parameters that describe its internal organization and structure. Interestingly, the inebriation level was positively correlated with the RSN density. The findings support our hypothesis for increased large-scale connectivity due to the GABAergic inhibition induced by alcohol. The elevated connectivity is pronounced in alpha, beta and theta bands in line with the distinct role of these frequencies at rest. Thus, it is suggested that despite the fact that reduced GABA transmission is linked to reduced functional connectivity in alcoholics, facilitated GABAergic inhibition following short-term inebriation is associated to pronounced brain functional connectivity. Finally, all networks under study exhibited small-world properties and differed significantly from ‘random’ ones computationally produced with the same characteristics.
